# Sphingosine 1-Phosphate Receptor 2 Induces Otoprotective Responses to Cisplatin Treatment

**DOI:** 10.3390/cancers12010211

**Published:** 2020-01-15

**Authors:** Wei Wang, Muthu K. Shanmugam, Ping Xiang, Ting Yu Amelia Yam, Vineet Kumar, Wee Siong Chew, Jing Kai Chang, Muhammad Zulfaqar Bin Ali, Marie J. Y. Reolo, Yee Xin Peh, Siti Nasuha Binte Abdul Karim, Andrew Y.Y. Tan, Takaomi Sanda, Gautam Sethi, Deron R. Herr

**Affiliations:** 1 Department of Pharmacology, Yong Loo Lin School of Medicine, National University of Singapore, Singapore 117600, Singapore; mdcww@nus.edu.sg (W.W.); phcsmk@nus.edu.sg (M.K.S.); phcxp@nus.edu.sg (P.X.); phcytya@nus.edu.sg (T.Y.A.Y.); phccws@nus.edu.sg (W.S.C.); changjk@u.nus.edu (J.K.C.); mjyreolo@gmail.com (M.J.Y.R.); yeexin92@hotmail.com (Y.X.P.); sitinasuha.ak@gmail.com (S.N.B.A.K.); 2 Department of Medicine, Yong Loo Lin School of Medicine, National University of Singapore, Singapore 119228, Singapore; csitakao@nus.edu.sg; 3 Department of Physiology, Yong Loo Lin School of Medicine, National University of Singapore, Singapore 117593, Singapore; phsvk@nus.edu.sg (V.K.);; 4 Neurobiology Programme, Life Sciences Institute, National University of Singapore, Singapore 117456, Singapore; 5 Cancer Science Institute of Singapore, National University of Singapore, Singapore 117599, Singapore; faqar90@gmail.com; 6 Department of Biology, San Diego State University, San Diego, CA 92182, USA

**Keywords:** cisplatin, ototoxicity, sphingosine 1-phosphate, hearing loss, reactive oxygen species, cochlea, acoustic startle response, auditory brainstem response, CYM-5478

## Abstract

Ototoxicity is a major adverse effect of platinum-based chemotherapeutics and currently, there remains a lack of United States Food and Drug Administration-approved therapies to prevent or treat this problem. In our study, we examined the role of the sphingosine 1-phosphate receptor 2 (S1P_2_) in attenuating cisplatin-induced ototoxicity in several different animal models and cell lines. We found that ototoxicity in S1P_2_ knockout mice is dependent on reactive oxygen species (ROS) production and that S1P_2_ receptor activation with a specific agonist, CYM-5478, significantly attenuates cisplatin-induced defects, including hair cell degeneration in zebrafish and prolonged auditory brainstem response latency in rats. We also evaluated the cytoprotective effect of CYM-5478 across different cell lines and showed that CYM-5478 protects neural-derived cell lines but not breast cancer cells against cisplatin toxicity. We show that this selective protection of CYM-5478 is due to its differential effects on key regulators of apoptosis between neural cells and breast cancer cells. Overall, our study suggests that targeting the S1P_2_ receptor represents a promising therapeutic approach for the treatment of cisplatin-induced ototoxicity in cancer patients.

## 1. Introduction

Cisplatin (*cis*-diamminedichloroplatinum (II), CDDP) is a widely used chemotherapeutic agent for the treatment of a number of cancers, including cervical, testicular, lung, neck, and head cancer [1,2,3,4,5,6]. However, cisplatin treatment can also lead to undesirable side effects, such as neurotoxicity and ototoxicity, which consequently cause dose-limiting changes in the drug treatment programs [7,8]. Ototoxic lesions were found to start manifesting in the early stages of cisplatin treatment, which subsequently leads to dose-dependent bilateral hearing loss [9,10]. Current literature suggests that cisplatin-mediated ototoxicity primarily involves the generation of reactive oxygen species (ROS) in target inner ear tissues [11]. This has led to the development of otoprotective strategies predominantly focusing on preventing ROS action and formation through antioxidant therapy [12]. However, antioxidant therapy is generally non-selective and can attenuate ROS only after it has already accumulated and has presumably mediated oxidative stress. These limitations are evident from the marginal efficacy of antioxidant treatment of cisplatin-induced neurotoxicity [13,14,15]. Thus, a potential specific and targeted treatment is essential for cisplatin-mediated ototoxicity.

Sphingosine 1-phosphate (S1P) is a potent bioactive signaling lipid that plays key roles in numerous cellular processes, such as cell apoptosis, differentiation, and proliferation [16,17]. S1P targets a family of five G protein-coupled receptors (GPCRs), S1P_1-5_, which have different patterns of expression across the body and affect various intracellular signaling pathways [18]. As a result, these receptors play diverse roles in most physiological processes and in many disease states, notably including inflammation, cancer, and differentiation [19,20]. The specific contribution of S1P_2_ to these processes is complicated, with evidence suggesting that this receptor can either positively [21,22,23,24] or negatively [25,26,27] regulate tumor growth and metastatic potential, presumably in a context-dependent manner. Interestingly, a novel mechanism was recently identified, whereby breast cancer cells may remodel their microenvironment by shedding exosomes containing constitutively active S1P_2_ [28], further illustrating the complexity of this receptor.

Previous work by our lab and others has shown that S1P_2_ is crucial for proper vestibular and auditory functions [29,30,31,32]. S1P_2_ is expressed in the cochlea and plays an important role in the maintenance of the cochlear hair cells [29]. Subsequently, we demonstrated that ROS accumulation precedes cochlear degeneration in *S1pr2^−/−^* mice and that S1P_2_ can attenuate ROS production in vitro by inhibiting nicotinamide adenine dinucleotide phosphate (NADPH) oxidase activity [33]. Since cisplatin-mediated ototoxicity is primarily the result of NADPH oxidase-generated ROS [2], we hypothesized that pharmacological activation of S1P_2_ would prevent this adverse effect.

Here, we report that ototoxicity present in the S1P_2_ knockout mice is dependent on ROS production and that S1P_2_ activation with a potent agonist, CYM-5478 [33], protects against cisplatin-mediated ototoxicity in rats and cisplatin-mediated hair cell death in zebrafish. In addition, we evaluated the selectivity of CYM-5478 and we showed that it protects neural cells but not breast cancer cells against cisplatin toxicity. We further identified key apoptosis regulatory proteins that are potentially involved in the CYM-5478 selective protection towards neural-derived cells. Cumulatively, these results further strengthen the rationale of targeting the S1P_2_ receptor to attenuate the ototoxic effects induced by platinum-based chemotherapeutics.

## 2. Results

### 2.1. Ototoxicity in S1pr2^−/−^ Mice is Dependent on ROS Production

**Figure 1:**
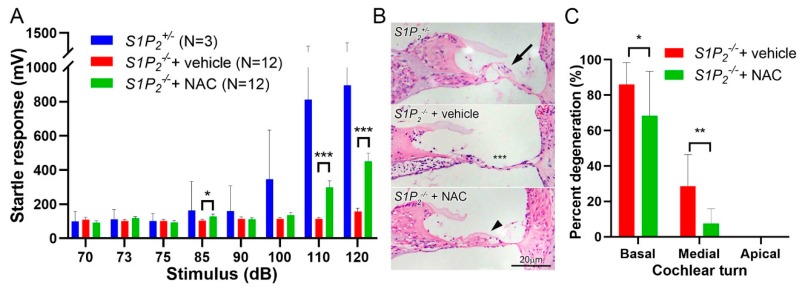
NAC attenuates cochlear degeneration in *S1pr2^−/−^* mice. (**A**) *S1pr2^−/−^* mice were treated with vehicle or NAC (1 g/kg/day, p.o.) between 1 and 5 weeks of age, then evaluated by ASR. A small cohort of *S1pr^+/−^* littermates were similarly evaluated at 5 weeks as a reference for normal ASR response. (**B**) Representative appearance of the organs of Corti in the basal turn of the cochlea at 6 weeks of age. *S1pr2^+/−^* mice uniformly present with intact organs of Corti containing the normal complement of three outer hair cells (arrow). *S1pr2^−/−^* mice were characterized by near-complete degeneration (asterisks). *S1pr2^−/−^* mice treated with NAC demonstrated decreased degeneration, although many organs of Corti were characterized by structural abnormalities (arrowhead). (**C**) Quantitation of the organ of Corti degeneration at 6 weeks of age. Degeneration indicates complete loss of the organ of Corti. (*n* = 12, * *p* < 0.05, ** *p* < 0.01, *** *p* < 0.001.).

Auditory perception was evaluated in mice using the acoustic startle response (ASR) assay, which measures sensorimotor reflex reaction to auditory stimulus. As previously described [33], S1P_2_ heterozygous mice (*S1pr2^+/−^*) demonstrated normal behavior with a conspicuous threshold response between 90 and 100 dB while there was a complete lack of a startle response in the *S1pr2^−/−^* knockout mice at 5 weeks of age (Figure 1A). Interestingly, *S1pr2^−/−^* mice treated with an antioxidant, N-acetylcysteine (NAC), demonstrated functional recovery of their hearing as evidenced by a significant startle response from 110 db onwards. Notably, the magnitude of the startle response was less than that of *S1pr2^+/−^* mice, consistent with an increased auditory threshold compared to wild-type mice. Furthermore, the protective effect of N-acetylcysteine (NAC) decreased by 6 weeks of age (data not shown), indicating that antioxidant administration was able to significantly delay, but not completely prevent, cochlear degeneration.

Histological analysis was carried out to assess the effects of NAC treatment on cochlear degeneration due to absence of S1P_2_. As expected, the progressive basal-to-apical cochlear degeneration was evident for the 6-week-old S1P_2_ knockout mouse, where there was a substantial organ of Corti loss in the basal turn without any notable degeneration at the apical region (Figure 1B). Treatment with NAC successfully reduced cochlear degeneration in the S1P_2_ knockout mouse, where the organ of Corti in the middle turn of the cochlea remained largely intact (Figure 1C) as compared to the untreated mouse, which had largely collapsed organs of Corti without inner and outer hair cells in the medial region (Figure 1B). The organ of Corti from each sample in the different treatment groups were categorized as either intact or degenerated before being quantified. There was an approximately 20% reduction in hair cell degeneration at the basal region and a significant three-fold reduction in the organ of Corti degeneration at the medial region after NAC treatment, which further underscores the impact of ROS in S1P_2_ knockout mouse ototoxicity. In addition, the results also highlight the important role of S1P_2_ in alleviating oxidative stress in the inner ear to maintain cochlear viability.

### 2.2. A Potent and Selective S1P_2_ Agonist, CYM-5478, Protects Against Cisplatin-Mediated Ototoxicity in Rats

**Figure 2:**
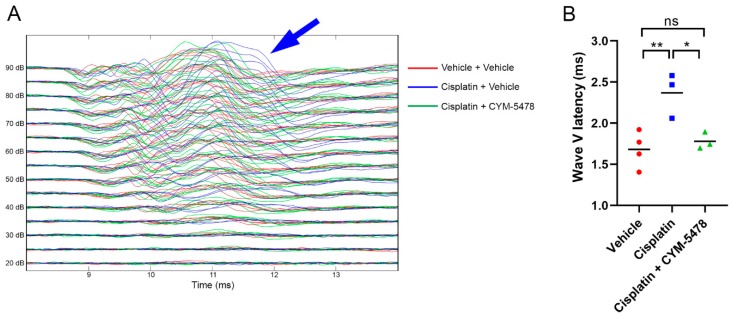
CYM-5478 prevents cisplatin-mediated ABR abnormalities. (**A**) Rats were treated with cisplatin for 3 weeks, with or without co-administration of CYM-5478, before hearing acuity was evaluated by ABR. Although all groups demonstrated similar response thresholds of ~35 dB, the cisplatin-only group exhibited increased waveform latency at all click intensities (arrow). Stimulus onset is at ~7 ms for all groups, including the time taken for sound to reach the ear. (**B**) Quantification of waveform V latency of 90 dB stimuli shown in (**A**). (*n* = 3–4, * *p* < 0.05, ** *p* < 0.01, ns = not significant.).

The effects of S1P_2_ activation on cisplatin-mediated ototoxicty in rats were then investigated using the auditory brainstem response (ABR) test, which measures the waveforms of an auditory evoked potential. Due to the use of a fairly conservative cisplatin dosing regimen, there was an absence of a threshold shift in the ABR after cisplatin treatment (Figure 2). However, there was an obvious increase in the wave V latency after cisplatin treatment as compared to the control. This cisplatin-induced shift in the wave V latency was ameliorated with the treatment of a potent and selective S1P_2_ agonist, CYM-5478 [33], which further emphasizes the protective role of S1P_2_ activation against cisplatin-mediated ototoxicity.

### 2.3. CYM-5478 Protects Against Loss of Hair Cell Viability in a Zebrafish Model for Ototoxicity

**Figure 3:**
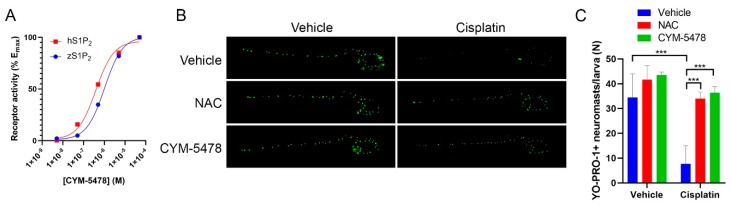
CYM-5478 attenuates cisplatin-mediated degeneration of zebrafish hair cells. (**A**) CYM-5478-mediated activation of the zebrafish homolog of S1P_2_ (zS1P_2_) was evaluated by the receptor-specific TGFα-shedding assay. Human S1P_2_ (hS1P_2_) was used as a reference. (**B**) Representative images of zebrafish larvae treated with cisplatin in the presence of NAC (1 mM) or CYM-5478 (20 μM). Viable neuromasts were labeled with YO-PRO-1. (**C**) Quantification of YO-PRO-1-positive neuromasts. (*** *p* < 0.001, *n* = 4).

The zebrafish lateral line is composed of hair cell-containing neuromasts that are functionally and structurally homogenous to cochlear hair cells. This system has been validated as a model to evaluate ototoxicity [34,35]. To determine whether CYM-5478 is suitable for use in zebrafish, we performed an in vitro receptor activation assay. This confirmed that CYM-5478 is an effective agonist for the zebrafish homolog of S1P_2_, albeit at a ~3-fold lower potency relative to human S1P_2_ (Figure 3A). Using immunofluorescence staining, we then investigated the effects of cisplatin treatment on the viability of the hair cells at the lateral line of the zebrafish and whether treatment with NAC or CYM-5478 has any protective effects (Figure 3B,C). Cisplatin led to a decrease in hair cell viability, which was ameliorated by both NAC and CYM-5478 as shown by the increase in neuromast labeling after NAC and CYM-5478 treatments (Figure 3B). Quantification of the immunofluorescence labeling indicates that both NAC and CYM-5478 treatment resulted in near-complete protection from cisplatin-mediated loss of neuromast viability (Figure 3C).

### 2.4. CYM-5478 Protects Neural Cells but Not Breast Cancer Cells Against Cisplatin Toxicity

**Figure 4:**
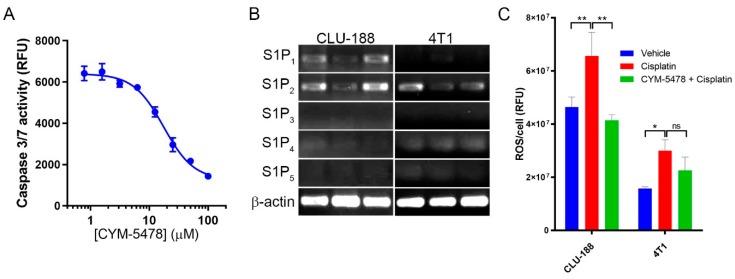
CYM-5478 attenuates apoptosis and oxidative stress in neural cells. (**A**) C6 glioma cells were treated with cisplatin (20 μM) for 24 h in the presence of increasing concentrations of CYM-5478, then evaluated for caspase 3/7 activity. (*n* = 6.) (**B**) cDNA prepared from CLU-188 mouse hypothalamic cells and 4T1 mouse mammary carcinoma cells was amplified with gene-specific primers. S1P_2_ is the most abundant S1P receptor present in both cell lines. (**C**) CLU-188 cells and 4T1 cells were treated with cisplatin (20 μM) overnight in the presence or absence of CYM-5478, then evaluated for ROS using the CellROX assay. (*n* = 5, * *p* < 0.05, ** *p* < 0.01).

We had previously demonstrated that the protective effect of CYM-5478 is able to reduce cisplatin-mediated cell death in C6 glioma cells, but the potency of this effect was not established [33]. To validate this result and establish effective concentrations for anti-apoptotic effects in vitro, we evaluated caspase 3/7 activity in cisplatin-treated cells (Figure 4A). Increasing concentrations of CYM-5478 caused a dose-dependent reduction in caspase 3/7 activity with a 50% effective concentration (EC_50_) of ~17.5 μM. To determine whether CYM-5478 is similarly cytoprotective against cells of non-neural origins, a cell viability assay was performed in a panel of cell lines in the presence of increasing concentrations of cisplatin, in the presence or absence of 20 μM CYM-5478. Our results showed that CYM-5478 reduced the toxicity of cisplatin (denoted by the significantly increased EC_50_ values) for many of the cell lines of neural origin but not for other cell types (Table 1). In fact, the sensitivity of all non-neural cancer cell lines to cisplatin was either unchanged or marginally increased in the presence of CYM-5478 (denoted by the significantly decreased EC_50_ values). This suggests that CYM-5478 should not desensitize tumor cells to cisplatin.

To identify whether selective attenuation of ROS generation contributes to the cell-type specificity of this response, the ROS content was evaluated in a murine neural cell line (CLU-188) relative to that of a murine breast tumor cell line (4T1). Both cell lines express S1P_2_ as their most abundant S1P receptor (Figure 4B). In both cell lines, cisplatin treatment stimulates a significant increase in ROS accumulation relative to vehicle-treated controls. However, only CLU-188 exhibits a significant attenuation of this effect with co-administration of CYM-5478 (Figure 4C). We further evaluated the effect of CYM-5478 on the cochlear neuroepithelial progenitor cell line, OC-k3. In contrast to the CNS-derived neural cell lines, there was no protective effect of CYM-5478 on cisplatin-induced cell death or ROS generation in OC-k3 cells (Supplementary Figure S1).

### 2.5. Potential Mechanisms of Action of Selective CYM-5478 Protection

**Figure 5:**
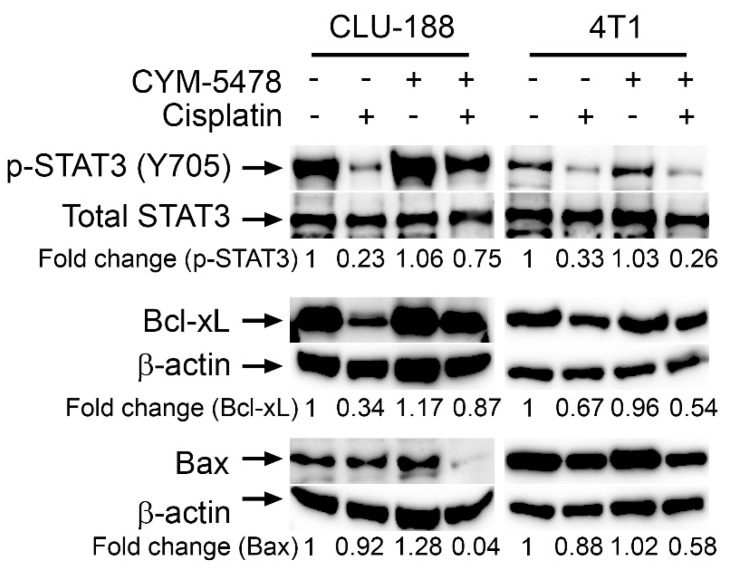
CYM-5478 promotes anti-apoptotic pathways in neural but not breast cancer cells. Cisplatin (20 µM) and CYM5478 (20 µM) either alone or in combination were treated for 24 h, after which western blot analysis was performed. CYM-5478 reverses cisplatin-induced suppression of anti-apoptotic phospho-STAT3 and Bcl-xL while inhibiting expression of pro-apoptotic Bax in CLU-188 mouse neural cells. CYM5478 had no effect on cisplatin-induced suppression of phospho-STAT3 or Bcl-xL, and did not suppress Bax in 4T1 mouse breast cancer cells.

To further investigate the potential mechanisms of action involved in the CYM-5478 selective protection towards neural-derived cells, western blot was carried out on a series of apoptosis regulating proteins in CLU-188 and 4T1 cell lines (Figure 5, Supplementary Figures S2–S13). Cisplatin treatment was found to decrease anti-apoptotic proteins, phospho- Signal transducer and activator of transcription 3 (p-Stat3) and B-cell lymphoma-extra large (Bcl-xL), in both CLU-188 and 4T1 cell lines. This response was attenuated by CYM-5478 in neuronal CLU-188 cells, but there was no effect of CYM-5478 in 4T1 breast cancer cells. In addition, CYM-5478 treatment resulted in a downregulation of pro-apoptotic Bcl-2-associated X (Bax) protein in cisplatin-treated CLU-188 cells, but not in 4T1 cells. This suggests that CYM-5478 selectively regulates apoptotic signaling pathways in neural-derived cells through the regulation of key apoptosis regulating proteins.

## 3. Discussion

Our cumulative results suggest that activation of the S1P_2_ receptor attenuates cisplatin-induced ototoxicity and hearing loss. We first studied the role of ROS production in the cochlear degeneration and hearing loss observed in S1P_2_ knockout mice. As previously reported, the S1P_2_ knockout mice presented profound deafness and cochlear degeneration [29] that is preceded by accumulation of ROS [33]. Here, we provide a causal link between these events by showing that treatment with an antioxidant, NAC, significantly attenuated both the hearing loss and structural degeneration observed in *S1pr2^−/−^* mice. 

Interestingly, while our results demonstrate that S1P_2_ plays a neuroprotective role in maintaining the integrity of cochlear neurons, recent studies suggest that this signaling pathway is similarly important in the development of sensory neurons. S1P_2_ was shown to be an important mediator of FGF-induced proliferation and a regulator of ERM proteins in an in vitro model for spiral ganglion progenitors [36,37]. This implicates S1P_2_ in the regulation of the cytoskeletal dynamic, membrane potential, and cell differentiation, suggesting a need to consider this pathway for the development of cochlear regenerative therapies [38]. 

Cisplatin was administered to rats to evaluate the otoprotective effect of CYM-5478 in mammals in vivo. In order to induce ABR threshold shifts in this assay, an aggressive dosing schedule is required that results in ~50% lethality [39]. We opted to use a more conservative schedule that better represents a clinically relevant dose that is sub-lethal but still results in profound neuropathy [40]. As expected, there was no detectable threshold shift; however, we observed a marked increase in the wave V latency, which was corrected after CYM-5478 treatment. This latency shift was similarly observed in a previous study of cisplatin-treated patients after two cycles of chemotherapy. These patients eventually developed hearing loss issues after subsequent additional chemotherapy treatment [41]. Wave V is generated primarily from the inferior colliculus, which is the main midbrain nucleus of the auditory pathway [42,43]. During the first few years of life, wave V latency was found to change regularly where it decreased rapidly in early childhood [43]. This decrease in wave V latency could be attributed to rapid growth in the axonal myelin density in the brainstem pathways and the cochlear nerve during early childhood [42,43]. In a previous study, the myelin sheaths of some type-I spiral ganglion cells in the cochlea were found to be detached from the perikaryon after cisplatin treatment [44]. Thus, it is possible that the cisplatin-mediated increase in wave V latency observed in our study indicates axonal myelin loss, leading to reduced conduction velocity that precedes sensorineural degeneration. The fact that this process is prevented by CYM-5478 suggests that S1P_2_ may exert its neuroprotective effects by preventing cisplatin-induced demyelination in the spiral ganglion, and possibly in the central nervous system.

Studying the viability of the hair cells on the lateral line of the zebrafish has been shown to be a reliable validated model to screen for drug-induced ototoxicity [34]. In our study, cisplatin treatment was shown to decrease hair cell viability, consistent with its ototoxic effects. Treatment with NAC was found to attenuate cisplatin-mediated hair cell death in the zebrafish model, demonstrating that ROS is indeed an important component of cisplatin-mediated ototoxicity. Similarly, activation of S1P_2_ with CYM-5478 markedly increased hair cell viability and ameliorated cisplatin-induced ototoxicity. The protective effect of CYM-5478 was comparable to the effect of NAC treatment, which further supports the model that activation of S1P_2_ mediates its otoprotective effect by attenuation of oxidative stress.

One of the primary complications associated with the use of antioxidants as otoprotective agents is their inherent lack of selectivity. Since many chemotherapeutics, including platinum compounds, exert their therapeutic effects at least in part though the generation of ROS, the administration of antioxidants may reduce chemotherapeutic efficacy [11,15]. Similarly, it was important to determine if S1P_2_ activation selectively inhibits cell death in neural cells or whether it is broadly cytoprotective. Thus, a variety of neural-derived cell lines and cancer cell lines were screened in the present study. We showed that CYM-5478 treatment selectively increased the viability of cell lines of neural origin while having a sensitizing or neutral effect in the other tumor cell lines. Taken together, these results suggest that CYM-5478 exerts selective protective effects and should not desensitize tumor cells to cisplatin treatment. In addition, previous studies have reported that S1P_2_ may act as a tumor suppressor for B-cell lymphomas as well as having anti-proliferative and anti-migratory effect in cancer cells [27,45]. Thus, besides attenuating ototoxicity, selective S1P_2_ agonist treatment may also potentiate the tumor cell inhibitory effects of platinum-based therapy. By contrast, a recent study has described positive effects of S1P_2_ on the proliferation and de-differentiation of neuroblastoma cells [46]. This suggests that S1P_2_ agonism may not be suitable for patients with neurological cancers. 

It was then postulated that the selective protection of CYM-5478 towards neural-derived cells was due to its effects on apoptosis regulating proteins. As expected, cisplatin treatment resulted in decreased levels of known anti-apoptotic proteins, such as phospho-Stat3 [47,48] and Bcl-xL [49,50,51]. CYM-5478 treatment, however, rescued phospho-Stat3 protein expression only in the neural cell line and not in the breast cancer cell line. This is particularly important since Stat3 and Bcl-xL are known to be involved in the chemoresistance of cancer cells [52,53]. Therefore, CYM-5478 should not affect Stat3/phospho-Stat3 expression in order for cisplatin to induce its chemotherapeutic effects in breast cancer cells [54]. In addition, CYM-5478 treatment was also found to inhibit Bax protein expression in the neural cell line. Bax is a pro-apoptotic protein and Bax inhibition was similarly found to protect hair cells against gentamicin- and TNFα-induced ototoxicity in previous studies [55,56]. Interestingly, while this manuscript was in preparation, an independent group identified a similar effect of S1P_2_ on Bax expression [57]. Using a genetic model in vivo, Li et al. demonstrated that enforced expression of S1P_2_ prevented nerve injury-induced upregulation of Bax in the spinal cord, and attenuated resulting allodynia.

In conclusion, this work supports the model that activation of S1P_2_ with a selective agonist can preferentially protect neural cells from chemotherapy-induced toxicity. Beyond the impact of this observation on the potential for preventing hearing loss, it is likely that S1P_2_ can provide a more broadly neuroprotective effect. Indeed, we recently reported that CYM-5478 can also reduce cisplatin-induced allodynia by attenuating glial activation in the dorsal root ganglion [58]. Future studies are necessary to determine if S1P_2_ activation would be similarly effective in other forms or neuropathy or in neurodegenerative disease. 

## 4. Materials and Methods 

### 4.1. S1P_2_ Knockout Mice

Genetically modified *S1pr2^−/−^* mice were purchased from Mutant Mice Resource and Research Centre (MMRC, Baltimore, USA). Mice were generated by deletion of the entire open reading frames of the gene encoding S1P_2_ by homologous recombination in a 129/SvJ, C57BL/6N mixed background as previously described [59]. The mice were housed in ventilated cages in groups with a maximum number of five in Centre for Life Science (CeLS) vivarium with a controlled temperature of (25 ± 3) °C and humidity of 55%. Mice were treated with NAC (Sigma-Aldrich #A7250) at 1 g/kg/day by supplementing drinking water with 0.67% NAC from one week old (before weaning) until six weeks old. Ad libitum intake was evaluated every two days and remained approximately 15 mL/100 g/day throughout the study. Control animals received regular drinking water. All housing and procedures were performed in accordance with the guidelines of NUS Institutional Animal Care and Use Committee (IACUC), Approval Number: 2014-00316.

### 4.2. Acoustic Startle Response (ASR)

ASR was performed using a startle response system (SR-LAB Startle Response System, San Diego Instruments, USA) within the Neuroscience Phenotyping Core in CeLS. Individually, mice were restrained inside a Plexiglas tube with a 40 mm inner diameter. Then, each subject was placed in a darkened and well-ventilated chamber where they were randomly exposed to defined variable sound intensities (65–120 dB). Responses were measured in arbitrary units with a force transducer and accompanying SR-Lab Software. Responses preceded by detectable motion in the restrained chamber prior to click stimulus were discarded. Twelve individual mice for every treatment group were tested.

### 4.3. Histology and Light Microscopy

Murine cochlea were evaluated as previously described [29]. Mice were sacrificed and the whole inner ear structures were removed intact. Extracted cochlea were fixed in 4% paraformaldehyde (Sigma Aldrich, St. Louis, MO, USA) overnight at 4 °C, decalcified with 4% hydrochloric acid (Sigma Aldrich, St. Louis, MO, USA)/4% formic acid (Sigma Aldrich, St. Louis, MO, USA) mixture for 24 h at 4 °C, and neutralized with diluted ammonia (Sigma Aldrich, St. Louis, MO, USA). The tissues were embedded in paraffin and sectioned to 5 μm. The sections were stained with alcoholic hematoxylin (Sigma Aldrich, St. Louis, MO, USA) and eosin (Sigma Aldrich, St. Louis, MO, USA) stain using standard protocols. Inner ear sections were evaluated under a 20× objective lens, and organs of Corti were categorized as intact or degenerated by a blinded researcher without prior knowledge of the treatment or mouse genotype.

### 4.4. ABR Methods

Female Sprague-Dawley rats weighing about 200 g (In vivos, Singapore) were used in the study. Animals were housed in groups of 2 per cage and maintained in a 12-h light/dark cycle (lights on and off at 7:00 and 19:00, respectively) in a temperature (22–24 °C) and humidity (45%–55%) controlled facility. Standard chow and water were provided ad libitum. The experimental procedures were approved by the IACUC at the National University of Singapore, approval #2017-00510. Cisplatin (#C2210000, Sigma Aldrich, St. Louis, MO, USA) was freshly prepared before each use by dissolving in sterile saline. In total, 3 mg/kg was administered intraperitoneally (i.p.) at 2 mL/kg to the rats once a week (days 0, 7, and 14). CYM-5478 (MolPort-004-121-217, MolPort, Latvia) was prepared weekly by dissolving in 100% dimethyl sulfoxide at 10 mg/mL then diluting in saline containing 0.1% TWEEN-20. 0.1 mg/mL immediately before i.p. administration at 1 mg/kg every day. Rats not receiving the indicated compounds were given an identical vehicle treatment. Auditory brainstem responses (ABRs) were assessed in a soundproof chamber on day 21 with procedures similar to those previously described [60]. Each rat was anesthetized by intraperitoneal injection of ketamine (75 mg/kg) and medetomidine (0.5 mg/kg). Body temperature was maintained with the help of a heating pad. Silver wires were inserted under the skin at the vertex and near the mastoid for each ear. The active electrode was placed at the ear receiving sound stimulation, the reference electrode at the vertex, and the ground electrode at the unstimulated ear. Recordings were made in a double-walled sound-isolating chamber (ETS-Lindgren, TX, USA). Sound stimuli were calibrated by recording pressure waveforms with a microphone (4939-A-011, Brüel & Kjær, Nærum, Denmark) placed 35 cm in front of the speaker (XT25TG30-04, Tymphany, Hong Kong). Each rat was then positioned with its stimulated ear where the microphone been placed. Electrode insertion and animal placement were carried out by a person blind to the treatment groups. Stimuli were alternating polarity clicks (0.1 ms duration, inter-click interval 70 ms, various intensities) and 16 kHz tones (12 ms duration, 1 ms linear rising and falling phases, inter-tone-interval 70 ms). The signal measured by the electrodes was filtered to lie between 300 and 5000 Hz and sampled at 20 kHz. For each stimulus type and sound intensity, the average of at least 500 responses was determined, with the number of responses used to estimate the average being the same across all sound intensities and treatment conditions.

### 4.5. TGFα-Shedding Assay

The zebrafish S1P_2_ homolog (zS1P_2_) was amplified by polymerase chain reaction from zebrafish larva cDNA using target-specific primers (left, 5′-GCCACCATGACTACTTGCCGTCTGTTTGCGG-3′; right, 5′-AACACAGGTAGTGCAGTCCTGC-3′) and was cloned into pcDNA3.1-V5-His-topo per the manufacturer’s protocol (Thermo Fisher Scientific, Inc., Waltham, MA, USA, #K480001). The TGFα-shedding assay was performed essentially as described [22,61]. Briefly, HEK293 cells were co-transfected with the indicated receptor expression construct, with Gα_q/i1_ chimeric protein, and with TGFα-alkaline phosphatase using Lipofectamine 2000 reagent (cat #11668019, Thermo Fisher Scientific, Inc., Waltham, MA, USA), collected by trypsinization, washed with phosphate-buffered saline, and seeded into 96-well plates in Hank’s Balanced Saline Solution (HBSS). Cells were stimulated with ligand for 1 h, then alkaline phosphatase activity was detected in cells and in the supernatant. Receptor activity (% shedding) was defined as alkaline phosphatase activity of the supernatant/total alkaline phosphatase activity (cells + supernatant). Data processing and statistical analyses were performed with GraphPad Prism 7.

### 4.6. Zebrafish Immunofluorescence Staining

Zebrafish larvae at 5 days post-fertilization were placed in 24-well plates at 4 larvae per well, then treated with indicated compounds in duplicate wells and incubated for 24 h. Compounds were removed by rinsing with E3 buffer and larvae were stained with YO-PRO-1 (Thermo Fisher Scientific Inc., Waltham, MA, USA, # Y3603) for 2 h per the manufacturer’s protocol. Larvae were euthanized sequentially with MS-222 and mounted on low melting agarose in a lateral position. Extent of neuromast degradation was photographed under an Axioplan 2 Imaging Fluorescent Microscope equipped with a Halogen Lamp (Olympus U-RFL-T). Automated quantification was performed using ImageJ. Images were inverted, converted to grayscale, processed with the threshold function to remove background fluorescence, and spot counted with analyze particles. All images were captured and processed with identical settings.

### 4.7. Cell Treatments with Cisplatin and CYM-5478

C6 rat glioma cells (ATCC #CCL-107), HEK293 cells (ATCC #CRL-1573), GT1-7 cells [62], CLU188 cells [63], SH-SY5Y cells (ATCC #CRL-2266), SK-N-BE2 cells (ATCC #CRL-2271), iNHA cells [63], MDA-MB-231 cells (ATCC #HTB-26), MDA-MB-436 cells (ATCC #HTB-130), MDA-MB-453 cells (ATCC #HTB-131), MCF7 cells (ATCC #HTB-22), 4T1 cells (ATCC #CRL-2539), BT474 cells (ATCC #HTB-20), CHO cells (ATCC #CCL-61), OVK18 cells [64], DU145 cells (ATCC #HTB-81), and HCCLM3 cells [65] were maintained as a monolayer culture on tissue culture dishes at 37 °C, 5% CO_2_, 100% humidity in Dulbecco’s modified Eagle’s medium (DMEM) supplemented with 10% heat-inactivated fetal bovine serum and antibiotics. SH-SY5Y cells were differentiated in the presence of 10 μM retinoic acid for 1 week [66]. OC-k3 cells [67] were maintained as a monolayer on tissue culture dishes at 33 °C, 10% CO_2_, 100% humidity in DMEM supplemented with 10% heat-inactivated fetal bovine serum, 50 U/mL of recombinant mouse interferon-γ, and antibiotics. S1P and CYM-5478 were solubilized with bovine serum albumin (0.1% final concentration) prior to treatment.

### 4.8. Reactive Oxygen Species (ROS) Assay

Cells were plated in 96-well plates at ~50% confluence, incubated overnight, then treated overnight with cisplatin in the presence of the indicated compounds. All conditions were vehicle-controlled (0.1% DMSO). Cells were treated with CellROX^®^ Orange reagent (Thermo Fisher Scientific Inc., Waltham, MA, USA, #C10443) per the manufacturer’s protocol and counterstained with Hoechst 33,342 (Thermo Fisher Scientific Inc. #62249). Cells were photographed with a 20X objective lens, 4 fields per well, 5 wells per condition on a Cytation 3 Cell Imaging Multi-Mode Reader (BioTek Instruments, Inc., Winooski, VT, USA). Quantification was performed using Gen5 software (BioTek Instruments, Inc. Winooski, VT, USA), by dividing the total integrated density of CellROX^®^ labelling by the number of cells per Hoechst staining. 

### 4.9. MTT Assay

Cell viability was determined by MTT assay as described [68]. Briefly, cells were seeded into 96-well plates at 20,000 cells/well, incubated overnight, and treated for 24 h with a dilution series of cisplatin in the presence or absence of CYM-5478 (20 μM). Cells were then incubated with 1 mg/mL MTT reagent for 90 min, washed carefully with PBS, solubilized with 4 mM HCl/0.1% NP40/isopropanol, and measured at 590 nm with a reference filter of 620 nm on a plate reader. All assays were performed with *n* = 6. Results are representative of at least 3 independent experiments.

### 4.10. Western Blot

Whole-cell extracts from vehicle or CYM5478- or cisplatin-treated cells were prepared in RIPA lysis buffer. Lysates were then spun at 10,000 rpm for 10 min to remove insoluble material and stored at −80 °C for later use. The protein content in the lysates was measured by Bio-Rad protein assay dye reagent concentrate (Bio-Rad, Des Plaines, IL, USA) and an equal quantity of protein was resolved on a 10% SDS gel. After electrophoresis, the proteins were electrotransferred to a nitrocellulose membrane, blocked with Blocking One (Nacalai Tesque, Inc., Kyoto, Japan blocking buffer, and probed with rabbit polyclonal antibodies to total STAT3 and mouse monoclonal antibodies against phospho-STAT3 (Tyr 705), Bax, Bcl-xL, and beta-actin (Santa Cruz Biotechnology, Dallas, TX, USA) overnight at 4 °C. The blot was then washed with TBS with 0.1% Tween-20 and exposed to anti-rabbit–horseradish peroxidase (HRP) conjugate and goat anti-mouse HRP (Sigma-Aldrich, St. Louis, MO, USA) antibodies for 1 h, and finally examined by Western Bright Sirius HRP substrate (Advansta). Images were captured using Chemidoc XRS+ imaging system and analyzed using Image Lab™ software (Bio-Rad, Des Plaines, IL, USA). 

### 4.11. Statistical Analyses

All tests for significance were performed by two-way ANOVA with Tukey’s multiple comparison test. Differences were considered significant when *p* < 0.05. 

## 5. Conclusions

In conclusion, our in vivo and in vitro experiments have shown that activation of the S1P_2_ with a selective agonist, CYM-5478, protects against cisplatin-mediated ototoxicity by promoting hair cell viability and inhibiting ROS formation. We also showed that administration of CYM-5478 preferentially increased the viability in cell lines of neural origin while having a neutral or sensitizing effect in tumor cell lines, all of which suggest that CYM-5478 should not affect the activity of cisplatin in tumor cells. Altogether, our results are consistent with a model that supports the targeting of S1P_2_ to treat cisplatin-mediated ototoxicity.

## Figures and Tables

**Table 1:** Sensitivity of cell lines to cisplatin in the presence of vehicle or 20 μM CYM-5478. Green indicates a significant decrease in sensitivity. Red indicates a significant increase in sensitivity. Values in bold indicate statistical significance (*p* < 0.05).

Cell Origin	Cell Line	EC_50_ Vehicle	EC_50_ CYM-5478	*p* Value
Neural-derived	C6	1.34	4.54	**<0.001**
	GT1-7	5.47	17.0	**<0.001**
	SK-N-BE2	4.06	7.44	**<0.001**
	CLU188	3.23	5.54	**0.00160**
	SH-SY5Y (undifferentiated)	6.13	8.35	0.203
	SH-SY5Y (differentiated)	14.1	25.3	**0.00830**
	iNHA	18.6	17.5	0.821
Breast cancer	MDA-MB-231	66.1	53.2	0.178
	MDA-MB-436	8.72	8.16	0.882
	MDA-MB-453	8.92	4.74	**<0.001**
	MCF7	57.9	36.7	**0.0429**
	4T1	8.16	4.09	**<0.001**
	BT474	24.4	18.4	0.0670
Ovarian cancer	CHO	10.36	9.12	0.358
	OVK18	9.64	13.9	0.0729
Prostate cancer	DU145	1.687	1.27	0.204
Liver cancer	HCCLM3	12.5	11.7	0.590

## Supplementary Materials

The following are available online at https://www.mdpi.com/2072-6694/12/1/211/s1, Figure S1: CYM-5478 does not protect cochlear neuroepithelial OC-k3 cells from cisplatin toxicity, Figure S2: Western blot of lysates from CLU188 cells with α-phospho-STAT3, Figure S3: Western blot of lysates from 4T1 cells with α-phospho-STAT3, Figure S4: Western blot of lysates from CLU188 cells with α-STAT3, Figure S5: Western blot of lysates from 4T1 cells with α-STAT3, Figure S6: Western blot of lysates from CLU188 cells with α-BCL-xl, Figure S7: Western blot of lysates from 4T1 cells with α-BCL-xl, Figure S8: Western blot of lysates from CLU188 cells with α-βactin corresponding to the α-BCL-xl blot, Figure S9: Western blot of lysates from 4T1 cells with α-βactin corresponding to the α-BCL-xl blot, Figure S10: Western blot of lysates from CLU188 cells with α-Bax, Figure S11: Western blot of lysates from 4T1 cells with α-Bax, Figure S12: Western blot of lysates from CLU188 cells with α-βactin corresponding to the α-Bax blot, Figure S13: Western blot of lysates from 4T1 cells with α-βactin corresponding to the α-Bax blot.
