# Supplementary material for: Sphingosine 1-Phosphate Receptor 2 Induces Otoprotective Responses to Cisplatin Treatment
Source: Cancers (Basel). 2020 Jan 15;12(1):211. doi: 10.3390/cancers12010211 (PMC7016659; doi:10.3390/cancers12010211)
Supplement: Supplementary file 1 [file cancers-12-00211-s001.pdf]

# Supplementary Materials:

# Sphingosine 1-Phosphate Receptor 2 Induces Otoprotective Responses to Cisplatin Treatment

**Wei Wang, Muthu K. Shanmugam, Ping Xiang, Ting Yu Amelia Yam, Vineet Kumar, Wee Siong Chew, Jing Kai Chang, Muhammad Zulfaqar Bin Ali, Marie J. Y. Reolo, Yee Xin Peh, Siti Nasuha Binte Abdul Karim, Andrew Y.Y. Tan, Takaomi Sanda, Gautam Sethi and Deron R. Herr**

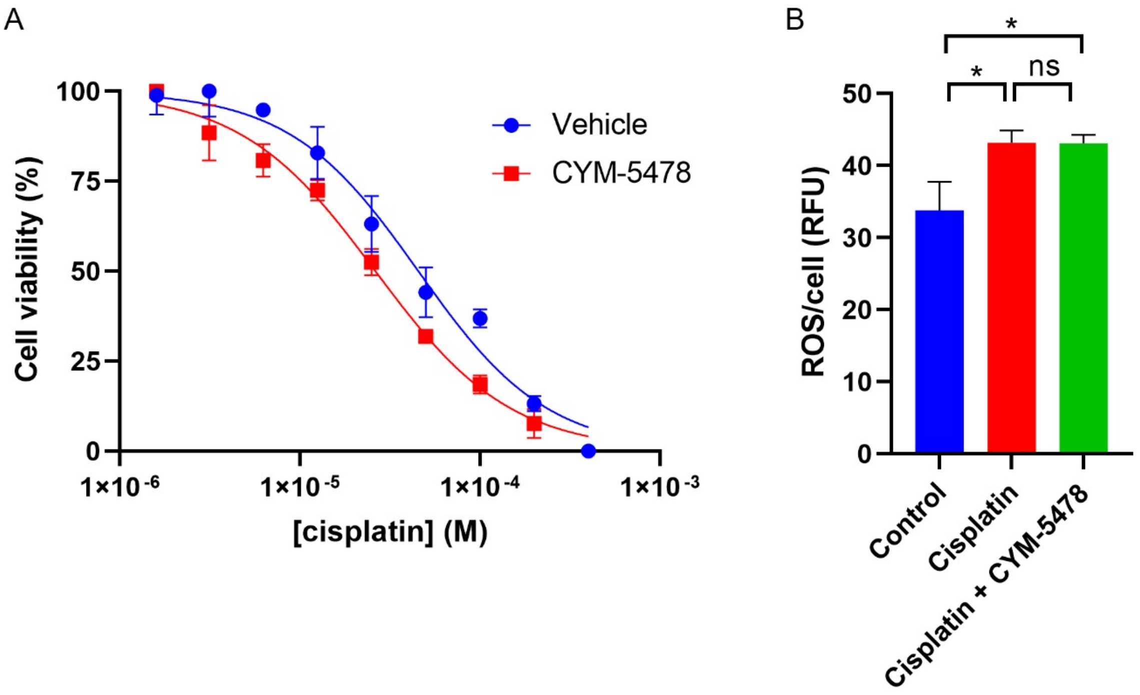


**Supplementary Figure S1.** CYM-5478 does not protect cochlear neuroepithelial OC-k3 cells from cisplatin toxicity. (**A**) OC-k3 cells were treated with increasing concentrations in the presence of vehicle or 20 μM CYM-5478 for 24 hours prior to evaluation of viability by MTT assay. No significant difference was observed between $EC_{50}$ values for vehicle and CYM-5478 groups. (**B**) OC-k3 cells were treated with vehicle, 20 μM cisplatin, or 20 μM cisplatin and 20 μM CYM-5478 for 24 hours prior to evaluation by CellROX assay. Cisplatin caused a ~25% increase in CellROX intensity relative to vehicle-treated controls. There was no significant difference between cisplatin alone and cisplatin + CYM-5478. N = 3, *$p < 0.05$, ns = not significant.

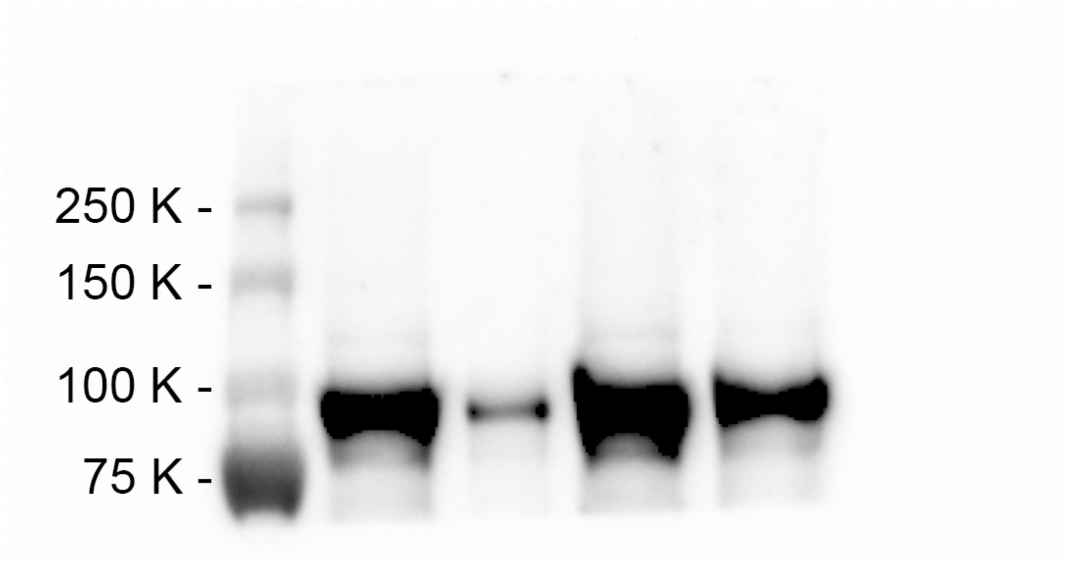


**Supplementary Figure S2.** Western blot of lysates from CLU188 cells with $\alpha$-phospho-STAT3.

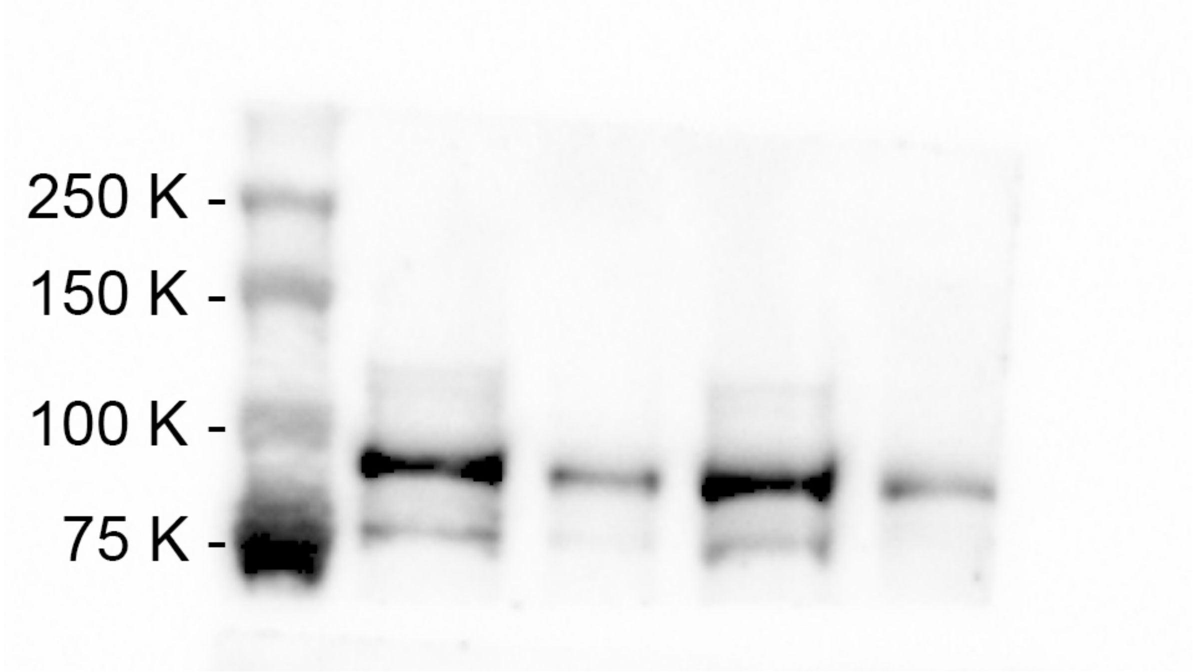


**Supplementary Figure S3.** Western blot of lysates from 4T1 cells with $\alpha$-phospho-STAT3.

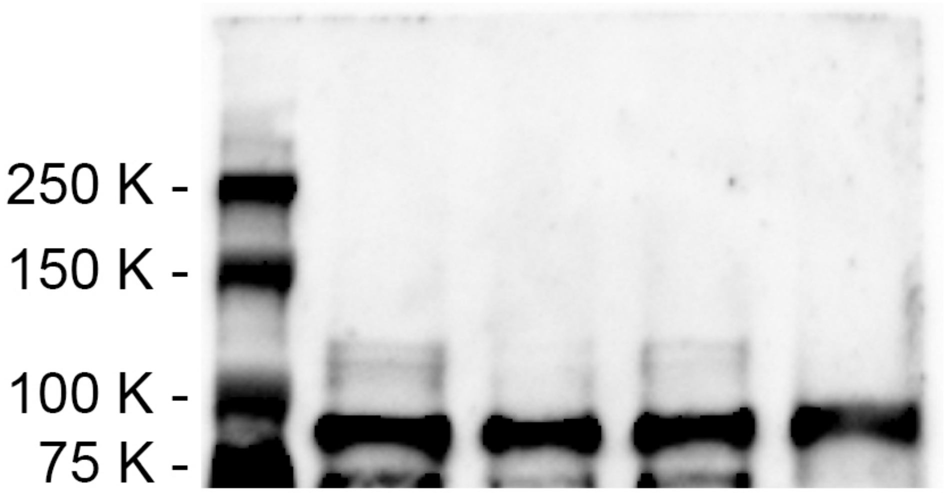


**Supplementary Figure S4.** Western blot of lysates from CLU188 cells with $\alpha$-STAT3.

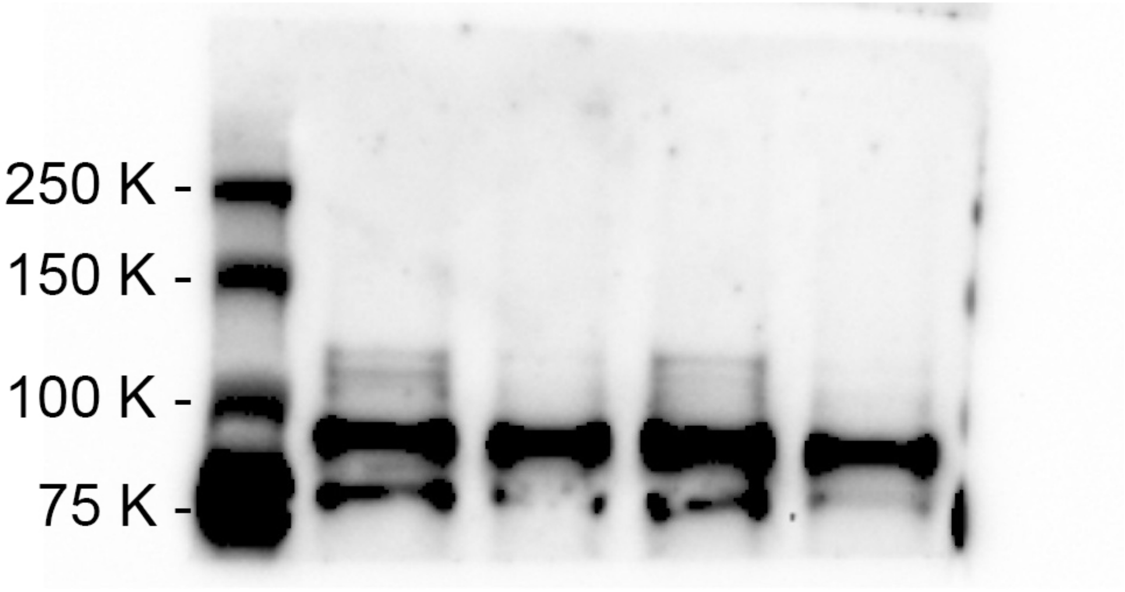


**Supplementary Figure S5.** Western blot of lysates from 4T1 cells with $\alpha$-STAT3.

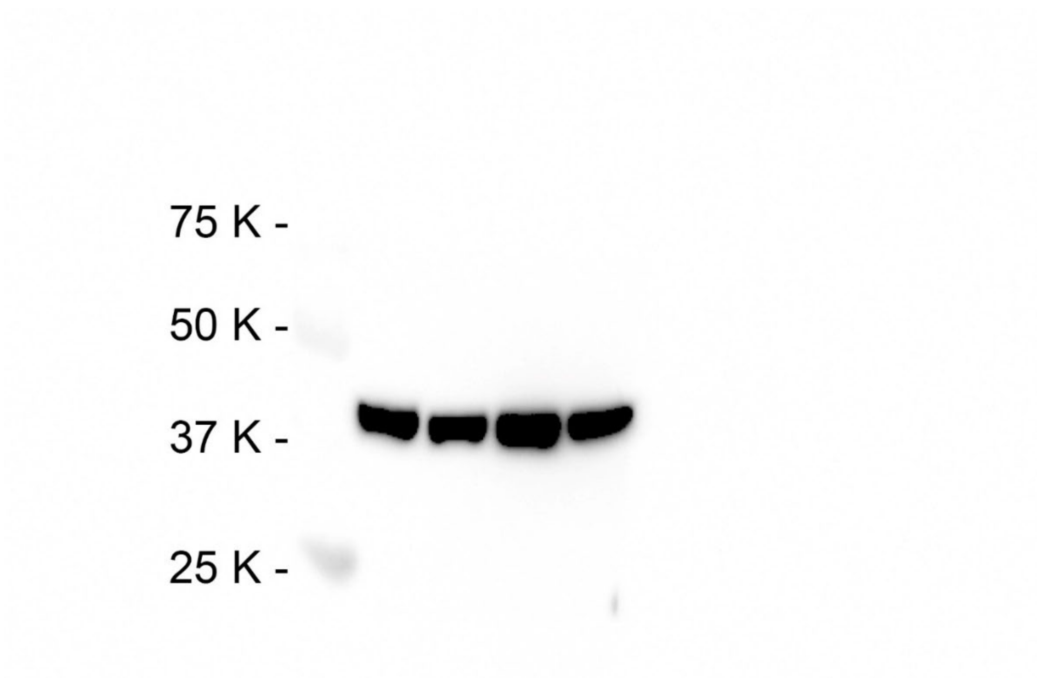


**Supplementary Figure S6.** Western blot of lysates from CLU188 cells with $\alpha$-BCL-xl.

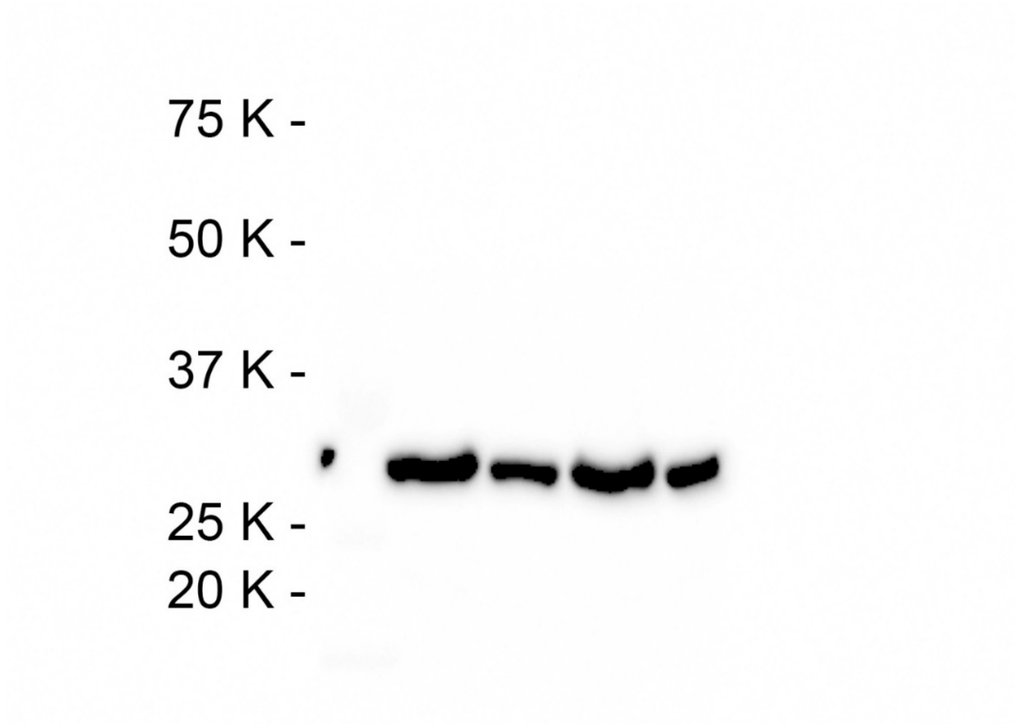


**Supplementary Figure S7.** Western blot of lysates from 4T1 cells with $\alpha$-BCL-xl.

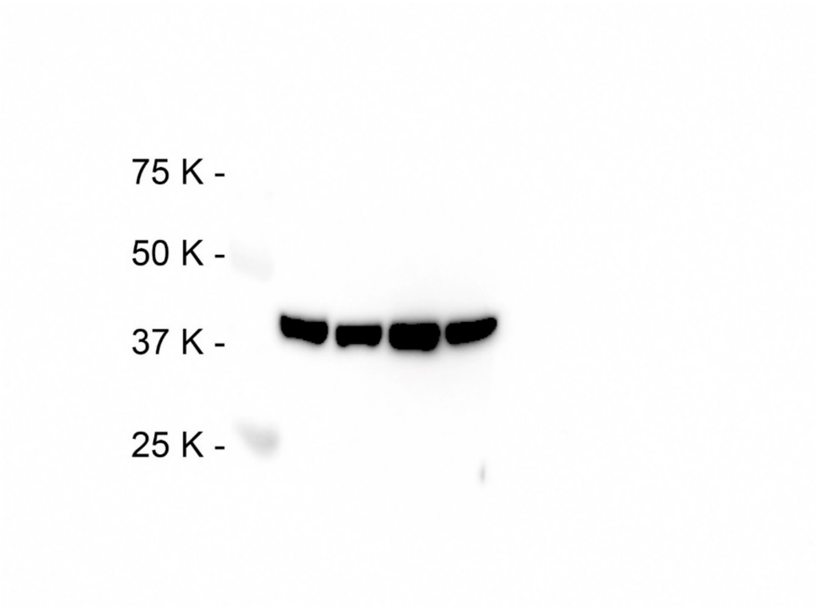


**Supplementary Figure S8.** Western blot of lysates from CLU188 cells with α-βactin corresponding to the α-BCL-xl blot.

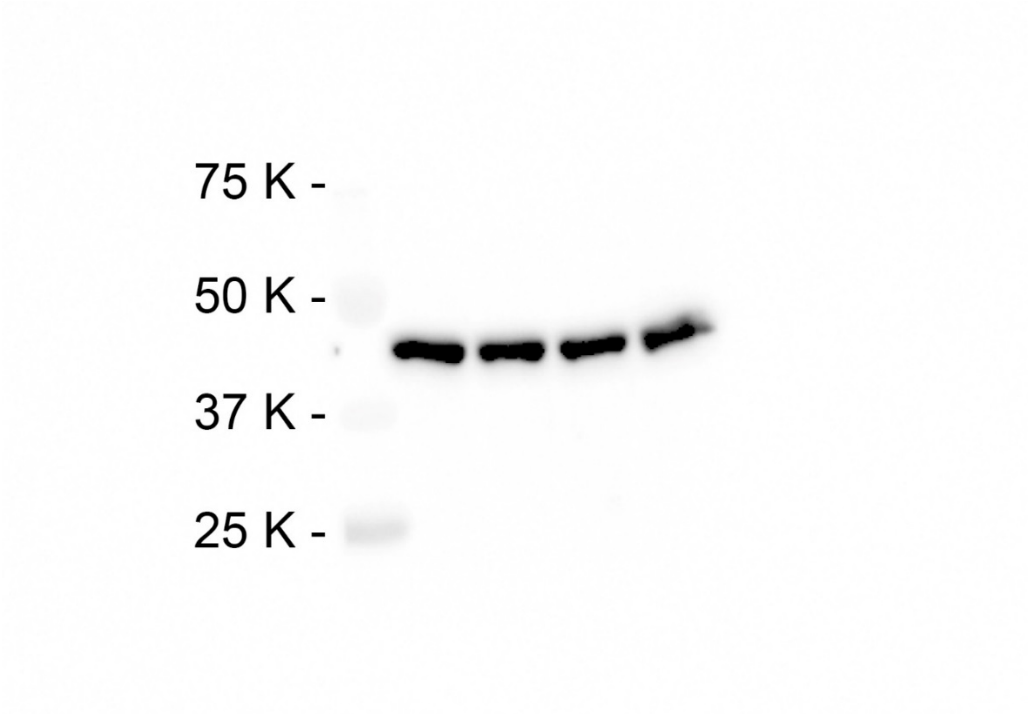


**Supplementary Figure S9.** Western blot of lysates from 4T1 cells with α-βactin corresponding to the α-BCL-xl blot.

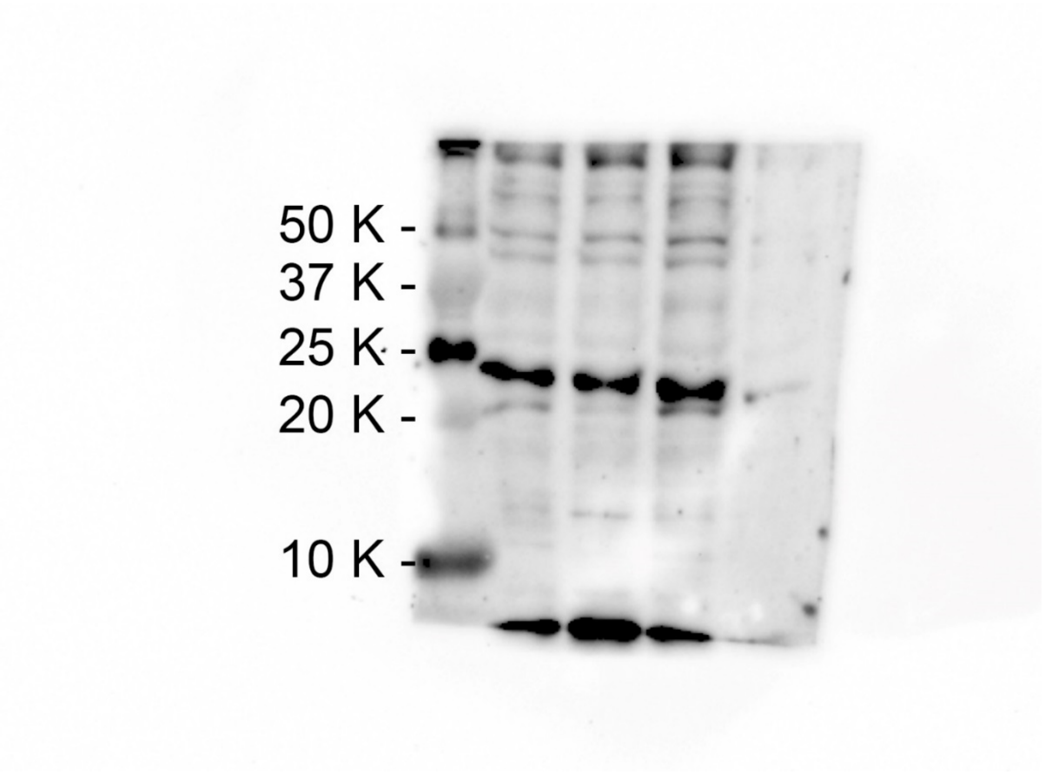


**Supplementary Figure S10.** Western blot of lysates from CLU188 cells with $\alpha$-Bax.

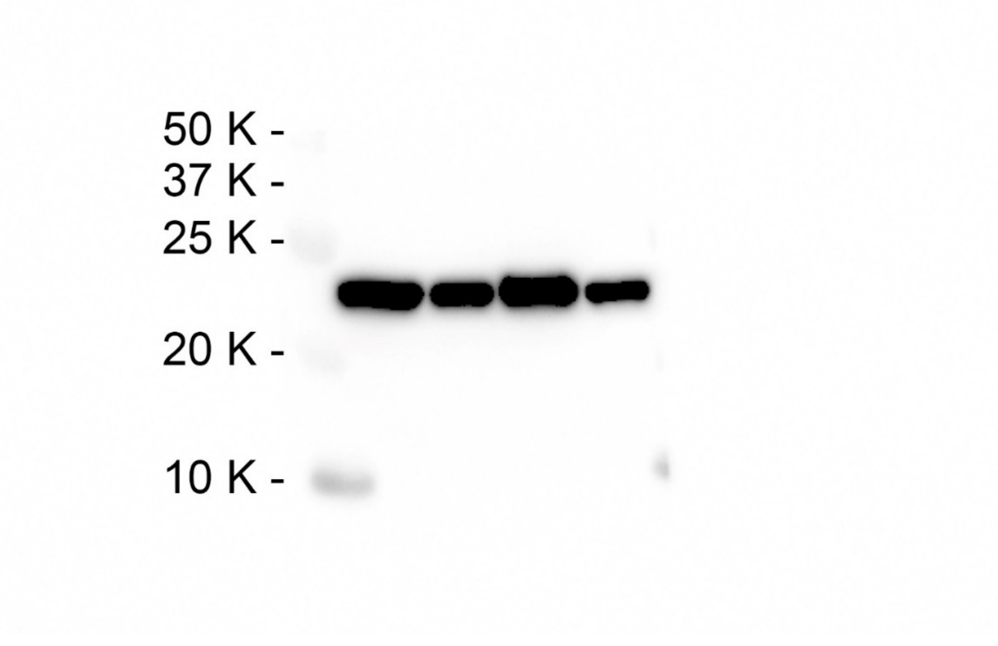


**Supplementary Figure S11.** Western blot of lysates from 4T1 cells with $\alpha$-Bax.

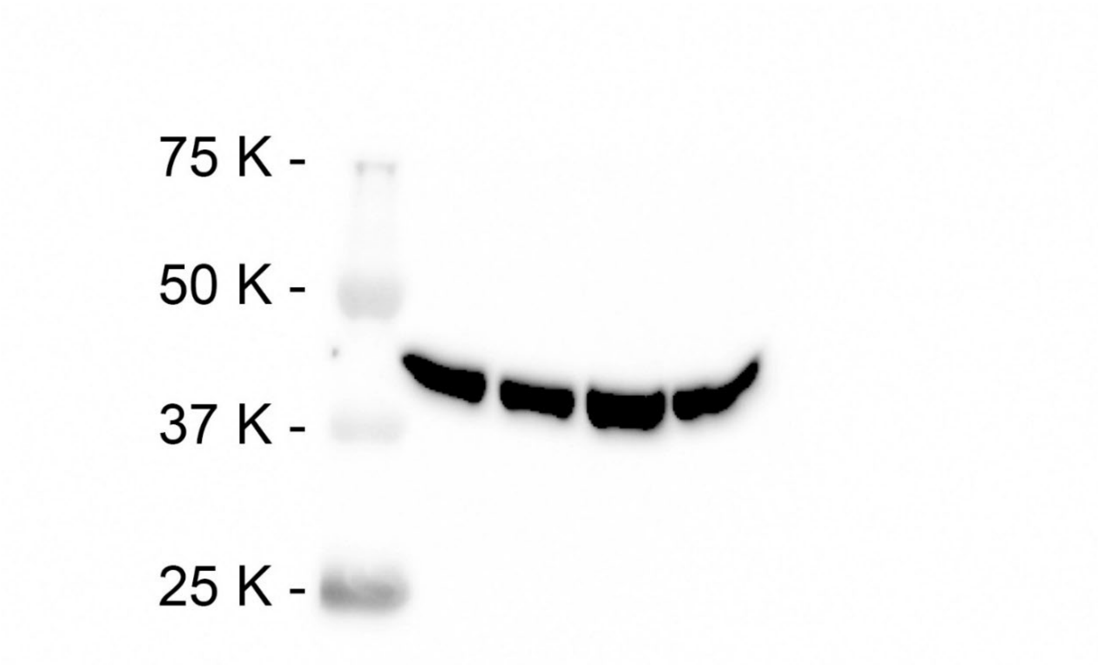


**Supplemental Figure S12.** Western blot of lysates from CLU188 cells with α-βactin corresponding to the α-Bax blot.

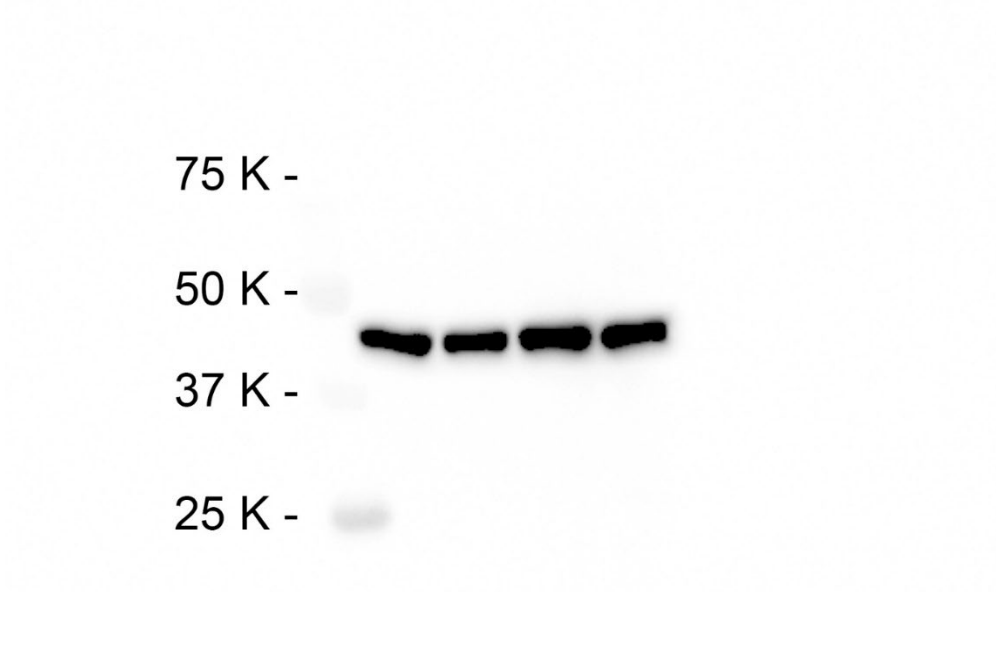


**Supplementary Figure S13.** Western blot of lysates from 4T1 cells with α-βactin corresponding to the α-Bax blot.
